# Depression and body mass index, a u-shaped association

**DOI:** 10.1186/1471-2458-9-14

**Published:** 2009-01-13

**Authors:** Leonore M de Wit, Annemieke van Straten, Marieke van Herten, Brenda WJH Penninx, Pim Cuijpers

**Affiliations:** 1 Department of Clinical Psychology and EMGO-Institute, VU University Amsterdam, van der Boechorststraat 1, 1081 BT, Amsterdam, The Netherlands; 2 Division of Social and Spatial Statistics, Statistics Netherlands, Heerlen, The Netherlands; 3 Department of Psychiatry and EMGO-Institute, VU University Medical Centre, Amsterdam, The Netherlands

## Abstract

**Background:**

Results of studies concerning the association between obesity and depression are conflicting. Some find a positive association, some a negative association and some find no association at all. Most studies, however, examine a linear association between Body Mass Index (BMI) and depression. The present study investigates if a nonlinear (U-shaped) trend is preferable over a linear trend to describe the relationship between BMI and depression, which means that both underweight and obesity are associated with depression.

**Methods:**

We investigated the existence of such a U-curve in a sample of 43,534 individuals, aged between 18–90 years, who participated in a cross-sectional study (Continuous Survey of Living Conditions) of physical and mental health in the general population of the Netherlands. We calculated linear and nonlinear (quadratic) ANOVA with polynomial contrast and curve fit regression statistics to investigate whether there was a U-shaped trend in the association between BMI and depression.

**Results:**

We find a very significant U-shaped association between BMI categories (underweight, normal, overweight and obesity) and depression (*p *≤ 0.001). There is a trend indicating a significant difference in the association between males and females (*p *= 0.05). We find a very significant U-shaped (quadratic) association between BMI (BMI^2^) and depression (*p *≤ 0.001), continuous BMI is not linearly associated with depression (*p *= 0.514).

**Conclusion:**

The results of this study give evidence for a significant U-shaped trend in the association between BMI and depression.

## Background

In recent decades, the association between obesity and depression has been examined in a considerable number of studies [1,2]. Both conditions are associated with increased risk of disability, reduced quality of life, increased mortality and co morbid conditions such as cancer, diabetes and coronary heart disease. The prevalence of both obesity and depression is very high, and both are associated with an enormous individual burden and huge economic costs [3].

Weight gain is for the most part influenced by decreased physical activity and increased intake of calorie-dense food. The development of obesity depends on genetic, metabolic and environmental factors [1,4]. Depression is caused by a combination of biological, psychological and social factors, and most researchers support vulnerability-stress models. In these models the development of a depressive disorder is triggered when a vulnerable (psychological and/or biological) individual experiences a life event or severe stress [5-9].

The exact underlying mechanism for the relationship between depression and obesity is not clear. Depression may cause obesity, for example through changing eating patterns or reduced physical activity [10-12]. But it is also possible that obesity may cause depression, for example through the negative body image which is the result of obesity [13]. Depression and obesity may also be caused by a third underlying factor. Socio demographic factors may moderate the association between depression and Body Mass Index (BMI, weight in kilograms divided by height in meters squared) [14].

Before causal pathways can be explored further, it is necessary to establish the exact pattern in which depression and obesity are associated with each other. Until now, three hypotheses have been suggested: a positive association between depression and obesity (higher depression is associated with more obesity) [15-17], a negative association (higher depression is associated with lower obesity), and no association [18-20].

From a psychiatric point of view, however, it could be expected that both overweight and underweight are associated with depression. According to the DSM-IV [21], in which the diagnoses of mental disorders are described, eating problems (eating too much or eating too little) and changed physical activity (increased or decreased) both constitute core symptoms of a major depressive disorder. Even though an increase in food intake might be more likely than a decrease in food intake, there is not much evidence to support this assumption. Furthermore, even though a decrease in food intake might occur less frequently, it might still affect large numbers of people since depression is such a prevalent disorder. Based on the assumption that food intake might decrease as well as increase, one would not expect a linear association between depression and obesity, but a U-curved association in which people with underweight and overweight report more depressive symptoms, compared to people with a normal weight. Although this seems an obvious association, we could find only few studies in which the existence of such a U-curve was tested [22,23]. The first study [22] only found a U-curve for the unadjusted data. The second study [23] found a U-curve when comparing normal weight, overweight and obese category, but they did not include an underweight category. Therefore, we decided to examine in a large population based sample whether we could find evidence for the existence of such a U-curve.

## Methods

### Study population

To monitor the physical and mental health of the general population of the Netherlands, a survey was carried out by the Statistics Netherlands (CBS). This continuous Survey of Living Conditions includes a representative sample of 10.000 individuals, each year. The randomization of the general population sample is conducted in two steps: Firstly, municipalities are randomly selected from 40 so called COROP areas (strata) in the Netherlands. Secondly, persons are randomly selected from the selected municipalities. The initial non-response rate varies each year between 35–40%. This cross-sectional study used a sample of all respondents aged between 18 and 90 years, who participated between the year 2001 and 2006. In total, 43,534 inhabitants of the Netherlands participated in this time period. All participants were interviewed at home by a trained interviewer. The interview consisted of a broad range of questionnaires evaluating aspects that included BMI and psychological well-being using Computer Assisted Personal Interviewing (CAPI). After the interview the respondents received a paper questionnaire. This paper questionnaire consists of more sensitive topics like (excessive) drinking and smoking and the presence of chronic diseases. It also contains the questions regarding depressive symptoms. The respondents filled in the paper questionnaire themselves after the interview and sent it back to Statistics Netherlands. The study survey was carried out according to the (ethical) policy of Statistics Netherlands (CBS) [24]. According to Dutch law there is no approval of the Medical Ethical Committee needed for surveys that are not aggravating for the respondents.

### Measures

Body Mass Index (BMI) was calculated as weight (kg) divided by height in meters squared (m^2^). Height and weight were self-reported. BMI was classified into four categories: Underweight (BMI < 18.5 kg/m^2^), Normal weight (BMI 18.5–24.9 kg/m^2^), Overweight (BMI 25.0–29.9 kg/m^2^) and Obesity (BMI > 30.0 kg/m^2^) [25]. BMI above 60 and below 14 was considered as an outlier and excluded.

Depressive symptoms were rated using the Mental Health Inventory (MHI), which is a 5 item subscale with 6 response categories each, of the Short Format-36 (SF-36), an extensive international standard for measuring common health status [26]. The MHI measures psychological health, so we calculated inverse scores of the MHI as an indication of depression symptoms and transformed them to a scale with a range 0 to 100 points, higher scores indicate an elevated level of depressive symptomatology. The MHI is a valid instrument for detecting mood disorders [27,28]. Socio demographic variables examined were: gender, age, ethnicity and level of education.

### Data analysis

All the analyses were conducted with SPSS 12.0.1 for Windows (SPSS Inc., Chicago, Illinois, USA). First we tested whether the observations of the socio demographic variables were associated with BMI, using Pearson χ^2 ^test.

Second, we investigated whether the association between BMI categories (underweight, normal weight, overweight and obesity) and depression was U-shaped. Therefore we conducted univariate linear and non linear ANOVA with polynomial contrast. Besides linear trends, this method also examines quadratic (U-shaped) trends [29]. The linear contrast compared the lowest with the highest BMI category and the quadratic compares both middle with the highest and the lowest BMI categories together [30].

We also conducted multivariate ANOVA with polynomial contrast for the BMI categories (underweight, normal weight, overweight and obesity) and depression, controlling for socio-demographic variables (gender, age, ethnicity, year of publication). Additionally, we tested whether the association between categorical BMI and depression was different for males and females. To test this question we included an interaction term for BMI and gender in our analysis (ANOVA with polynomial contrast).

We investigated whether the association between continuous BMI and depression was linear (BMI) or quadratic (U-shaped), using curve fit regression statistics [31]. This method calculated linear and non linear regression statistics including quadratic (U-shaped) trends. The method evaluates whether there is a deviation from linearity and if this is indeed present, it examines whether a quadratic trend is involved. Additionally, these analyses were repeated for the association between continuous BMI and depression in the different age and gender subgroups.

## Results

Of the total sample (44,374), 4,574 (10.3%) persons were considered obese, 15,230 (34.3%) were overweight, 22,660 (51.1%) had a normal weight and a total of 800 (1.8%) had underweight, while 1,100 (2.5%) had missing values. The mean MHI score of the total sample was 21.2 (SD: 15.4; Range: 100), 10,349 (23.3%) persons had missing values.

Table 1 shows the distribution of the socio demographic variables (gender, age, ethnicity, level of education, year of the study) and their association with BMI. As expected, the χ^2 ^analysis for depression and the socio demographic variables showed all significant (*p *< 0.05) associations.

**Table 1:** Selected characteristics of the sample, and the association with BMI^b)^

**Variable**	**Value**	**Total (N)**	**%**	**Under-weight (%)**	**Normal weight (%)**	**Over-weight (%)**	**Obese (%)**	***P***
**Gender**	Male	21181	48.65	1.11	47.70	41,7	9.49	0.000
	Female	22083	50.72	2.55	56.86	28.97	11.61	
**Age**	20–29	6027	14.27	4.05	70.42	20.97	4.56	0.000
	30–39	8649	20.47	1.72	56.89	32.18	9.21	
	40–49	8526	20.18	1.15	53.00	35.16	10.68	
	50–59	7908	18.72	0.81	43.56	41.79	13.66	
	60–69	5590	13.23	0.91	39.84	44.35	10.82	
	70+	5274	12.48	2.01	43.08	42.78	12.53	
**Education**	Preschool only	6938	15.93	1.90	40.83	40.28	16.98	
	High school lower level	6851	15.74	1.72	46.08	38.50	13.69	0.000
	High school medium level	3347	7.69	2.38	55.46	32.38	9.78	
	High school high level	15799	36.29	2.00	54.59	34.29	9.12	
	University/college	9668	22.21	1.46	60.36	31.71	6.46	
**Ethnicity**	Dutch	37481	86.66	1.79	52.29	35.47	10.45	0.000
	Foreign/western	3392	7.84	2.09	54.57	33.37	9.96	
	Foreign/non-western	2390	5.52	2.47	50.59	33.64	13.31	
**Year of survey**	2001	6861	15.86	1.81	53.29	35.48	9.63	0.009
	2002	6942	16.05	2.03	53.13	35.00	9.84	
	2003	6896	15.94	1.97	51.60	35.77	10.66	
	2004	7897	18.25	1.77	51.64	35.49	11.09	
	2005	7559	17.47	1.79	53.18	34.13	10.09	
	2006	7109	16.43	1.74	51.48	35.39	11.38	

^a^) All associations were examined with χ^2 ^analyses

^b) ^BMI Categories: Underweight (14–18.5 kg/m^2^), Normal weight (18.5–25 kg/m^2^), Overweight (25–30 kg/m^2^), Obesity (30–60 kg/m^2^).

### U-shaped association

**Figure 1:**
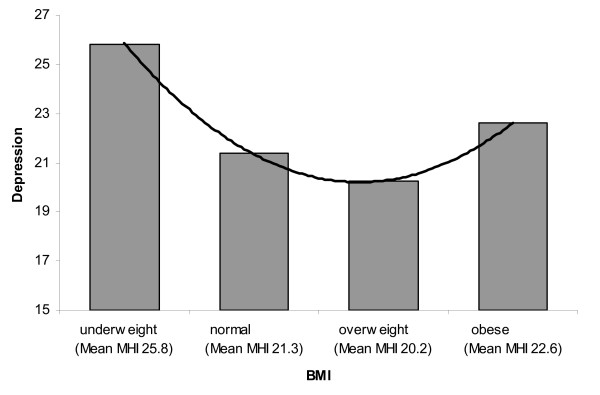
**U- curved association between BMI and depression**.

Results of the polynomial trend analyses indicated a significant positive quadratic effect between categorical BMI and depression (*p *≤ 0.001) and a contrast estimate of 3.43. After controlling for socio demographic variables (gender, age, ethnicity, level of education), the positive quadratic effect remained significant (*p *≤ 0.001) and a contrast estimate of 2.78. This means that the depressive score shows a positive quadratic trend in the association with the four BMI categories. So these data indicate a U shaped trend in the association between depression and BMI. Figure 1 shows the mean MHI scores over the BMI categories. Results of the interaction test for gender, showed a trend indicating a possible difference in the association between males and females (*p *= 0.05).

Furthermore we conducted confirmative analyses with the continuous indicator of BMI, expecting quadratic association. Results showed a significant quadratic association (β = 0.430, *p *≤ 0.001) and a non significant linear association (β = -0.004, *p *= 0.514) between BMI and depression. Table 2 gives an overview of quadratic regression statistics for quadratic association between BMI and depression in the subgroups age and gender, which were all significant (*p *≤ 0.001).

**Table 2:** The regression statistics for the U-shaped association between continuous BMI and depression.

**Variable**	**Value**	**β**^a)^	**Standard Error**	***P***
**All**		0.43	0.00	0.000
**Gender**	Male	0.38	0.00	0.000
	Female	0.26	0.00	0.000
**Age**	20–29	0.33	0.01	0.001
	30–39	0.53	0.01	0.000
	40–49	0.33	0.01	0.000
	50–59	0.53	0.01	0.000
	60–69	0.58	0.01	0.000
	70+	0.33	0.01	0.001

^a)^We report the squared data; a positive value indicates that past a certain point of BMI, the level of depression increases (U-curve).

## Discussion

The goal of this study was to explore if there is a U-shaped trend in the association between BMI and depression. In this community based sample of 43,534 participants we indeed found evidence of such a positive U-curve. Both BMI categories and BMI continuous BMI are nonlinear (U-shaped) associated with depression. We demonstrated that both obesity and underweight are associated with an increased level of depression, even after controlling for various socio demographic variables. Furthermore we found a difference in the association between men and women.

The results of this study emphasize the importance of distinguishing between (the four) different BMI categories when we investigate the nature of the association with depression. The underweight population should be examined as a distinct category because there could be a higher level of depression.

For example, in a previous study that focused on the association between obesity and depression, comparisons of depression levels were made between a sample of obese and a sample of non-obese subjects which included the underweight sample [32]. Our findings lead us to conclude that if one compares the levels of depression between the obese and non obese groups in this way, the results might be less significant because of the risks of high levels of depression in the underweight group. According to the DSM-IV, depression is associated with both increased and decreased food intake, and increased or decreased physical activity [21]. Therefore it seems logical that increased levels of depression are associated with obesity and also with underweight. It is notable that underweight was much less prevalent then overweight and obesity. A possible explanation might be that that decreased food intake is less prevalent then increased food intake as a symptom of depression. However these prevalences are not related to the existence of a U-curve and therefore do not affect our results.

Most studies focus on linear- (positive, negative) or no trends in the association between obesity and depression. Those studies investigate whether depression increases or decreases at higher levels of BMI [2].

There are studies that put forward the fact that a subset of the depressed is actually losing weight as a possible reason why the association between obesity and depression is not found in many studies [13]. Our study shows that this is indeed a plausible consideration.

The first strength of our study is that it contains a large sample of participants (44,374) and a broad variety of socio demographic variables, which we could use as covariates. The second strength is that the sample is random conducted in the Netherlands, which makes the results generalizable for the general population.

There were also several limitations. For the assessment of depression symptoms we used, the MHI. It is a valid instrument to measure depressive symptoms [27,28], but can not be used as a diagnostic instrument for depression. Furthermore, there were a considerable number of missing values on the MHI (23.3%) and BMI (2.5%) score which might have influenced the results. Especially if respondents suffering from depressive symptoms did not report the MHI score and people with underweight or obesity did not report their BMI score could that have caused bias in the results of this study. For the assessment of BMI we used self reported data. People tend to underestimate their weight and overestimate their height, this could jeopardize the validity of the results [33,34]. Furthermore the sample had a considerable amount of non-response (35–40% each year). This could possibly cause bias in the generalizability of the results when for instance the non response is larger among respondents with obesity, underweight or depression. Like many other studies concerning the association between BMI and depression, this study is cross-sectional. Therefore we can't explore the onset and causality and the reciprocal effects in the association between depression and BMI. Longitudinal studies are needed to examine the course of the depression and possible effect on BMI. Depression is known to appear in different episodes during a life span, so we need data on life time prevalence to study the association.

## Conclusion

We conclude that our findings clarify the nature of the association between BMI and depression. We found a U-shaped trend in the association. Longitudinal and experimental studies are needed to explore possible explanations of the relationship and the direction of causal relationships between BMI and depression.

## Competing interests

The authors declare that they have no competing interests.

## Authors' contributions

LdW wrote the first draft and the revisions of the manuscript. LdW and MvH conducted the analyses. AvS, PC and BP critically read each draft and contributed to the further drafting and revisions of the manuscript.

## Pre-publication history

The pre-publication history for this paper can be accessed here:


